# On-Surface Synthesis of Ligands to Elaborate Coordination Polymers on an Au(111) Surface

**DOI:** 10.3390/nano11082102

**Published:** 2021-08-19

**Authors:** Elie Geagea, Judicael Jeannoutot, Louise Morgenthaler, Simon Lamare, Frank Palmino, Frédéric Chérioux

**Affiliations:** FEMTO-ST, CNRS, Université de Franche-Comté, 15B Avenue des Montboucons, CEDEX, 25030 Besancon, France; elie.geagea@femto-st.fr (E.G.); judicael.jeannoutot@femto-st.fr (J.J.); louise.morgenthaler@yahoo.fr (L.M.); simon.lamare.pro@hotmail.fr (S.L.); frank.palmino@univ-fcomte.fr (F.P.)

**Keywords:** scanning tunnelling microscopy, on-surface synthesis, coordination polymers

## Abstract

On-surface metal-organic polymers have emerged as a class of promising 2D materials. Here, we propose a new strategy to obtain coordination polymers by transforming supramolecular networks into coordination polymers by surface-assisted cyclo-dehydrogenation of organic building blocks. All nanostructures are fully characterized by using scanning tunneling microscopy under ultra-high vacuum on a gold surface. We demonstrated that the balance between molecule-molecule interaction and molecule-substrate interaction can be drastically modified by a strong modification of the geometry of the molecules thanks to a thermal annealing. This new way is an efficient method to elaborate on-surface coordination polymers.

## 1. Introduction

During the past two decades, self-organization of molecular materials on surfaces has been widely investigated in the case of organic molecules and molecular objects deposited on different kind of substrates [1,2,3,4,5]. More recently, on-surface synthesis has emerged to provide the fabrication of covalent nanostructures [6,7,8,9,10,11,12,13,14,15,16,17,18,19,20]. All these nanostructures have been obtained by bottom-up approaches and their structure are atomically-precise, as proven by scanning probe microscopies, that can be used for great potential applications in molecular electronics, spintronics, energy, catalysis and other fields. Among all types of nanostructures, coordination polymers have attracted attention because coordination bonds are reversible, leading to the possibility to self-reparation by bond scission and reformation in order to achieve the formation of defect-free nanostructures [21,22,23,24,25]. These polymers can be directly obtained on crystalline substrate by the deposition of polyfunctional organic linkers that are able to coordinate adatoms of the surface or co-deposited metallic atoms in ultra-high vacuum. These organic linkers are able to coordinate metallic atoms or cations without any further action, because their coordination sites are in-the-plane and close to the underlying surface. The formation of the surface-confined coordination polymers is mainly governed by the balance between molecule-molecule and molecule-surface interactions, bond strength, reversibility of bond formation, precursor diffusion and their surface-concentration [2]. Finally, the nature of the metal atoms plays a key-role in the formation of the coordination polymers [21]. By tuning all these parameters, many surface-confined coordination polymers have been successfully achieved [20,21,22,23]. However, in most of cases, the underlying surface does not modify the ability of the organic linkers to coordinate metal centers. As on-surface chemistry gives the opportunity to create new building blocks only accessible when they are adsorbed at the surface, we propose to transform an organic molecule into an organic linker by surface-assisted reaction.

Here, we report a surface-assisted reaction that transforms organic molecules involved in large extended 2D-supramolecular networks into organic linkers being able to coordinate adatoms of the surface to give 1D coordination polymers. Experiments were performed on an Au(111) surface in ultra-high vacuum (UHV). All adsorbates are fully characterized by scanning tunneling microscopy that provides images with submolecular resolution. By using a surface-assisted cyclodehydrogenation reaction, we switch a purely 2D supramolecular network to coordination polymer by a controlled flattening of this organic molecule that gives it the ability to coordinate metal adatoms.

## 2. Materials and Methods

### 2.1. Synthesis

All reagents were purchased from Sigma-Aldrich, except Pd(PPh_3_)_4_ which was purchased from Strem chemical and used as received. The silica gel used for column chromatography was purchased from Merck. The deuterated NMR solvents were purchased from Euriso-top. The NMR spectra were recorded using a Bruker AC-300 MHz spectrometer. The two molecules, 10,10′-di-(4″-cyanophenyl)-9,9′-bianthryl (CPBA) and 10,10′-di-(4″-pyridyl)-9,9′-bianthryl (PBA) were synthesized by a procedure adapted from the literature [26,27]. Basically, 10,10′-dibromo-9,9′-bianthryl, 4′-cyanophenyl boronic acid pinacol ester or 4′-pyridyl boronic acid pinacol ester, cesium carbonate and Pd(PPh_3_)_4_ as a catalyst were dissolved in tetrahydrofuran. The resulting white solid was purified by column chromatography (silica gel, cyclohexane/dichloromethane 1:1) to give a white solid [28].

### 2.2. STM Experiments

The first step consists of the preparation of the Au(111) surface by argon ions sputtering cycles at 1.2 kV followed by thermal annealing at 673 K. This takes place inside a preparation chamber maintained under UHV conditions with a base pressure lower than 2 × 10^−10^ mbar and coupled to an Omicron variable temperature Scanning Tunneling Microscope (Omicron VT-STM XA). Once the cleanness of Au(111) surface is confirmed by analysis of STM images, we proceed to the deposition of molecules by thermal sublimation from a quartz crucible with corresponding temperature of 458 K for PBA and 468 K for CPBA. During deposition the substrate was held at room temperature then transferred and cooled to 110 K on the STM stage for acquiring images. Each image process was carried out using SPIP 6.7.7 (Digital Surf, France) software.

## 3. Results

**Figure 1:**
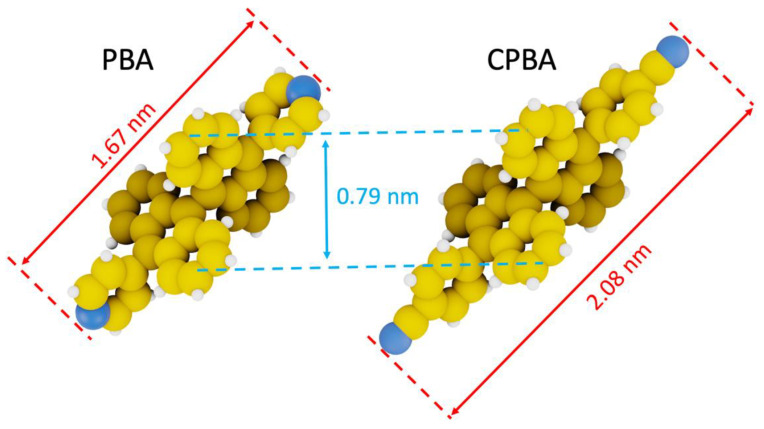
CPK models of 10,10′-di-(4″-pyridyl)-9,9′-bianthryl (PBA) and 10,10′-di-(4″-cyanophenyl)-9,9′-bianthryl (CPBA) molecules respectively.

We synthesized two molecules, 10,10′-di(4″-pyridyl)-9,9′-bianthryl (PBA) and 10,10′-di(4″-cyanophenyl)-9,9′-bianthryl (CPBA) respectively, that contain two aryl groups covalently bound to the 10 and 10′ positions (Figure 1) of a bianthryl core, respectively. We chose cyanophenyl and pyridyl groups as lateral groups because they are involved in the formation of supramolecular networks on various kinds of surface even at room temperature [5,29]. The distance between the extremities of anthracenly rings of these two molecules is 0.79 nm and their length is 1.67 and 2.08 nm for PBA and CPBA, respectively (Figure 1).

### 3.1. Supramolecular Self-Assembly on an Au(111) Surface

**Figure 2:**
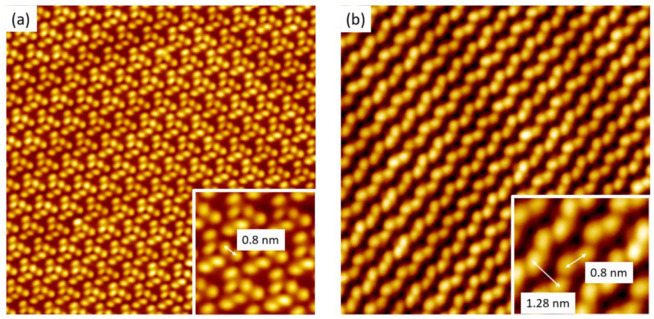
STM images of self-assembled molecules on Au(111) surface. (**a**) PBA supramolecular network (Vs = −1.5 V, It = 10 pA, 30 nm × 30 nm, inset 4 nm × 4 nm) (**b**) CPBA supramolecular network (Vs = 1.5 V, It = 10 pA, 20 nm × 20 nm, inset 3 nm × 3 nm).

PBA and CPBA molecules were deposited by thermal sublimation under UHV on an Au(111) substrate maintained at room temperature. Molecules were deposited in different coverage ratios but never exceeding the one monolayer threshold. As revealed in STM images recorded at 110 K, no isolated molecule was observed but only large extended 2D islands. Indeed, despite the amounts of molecules deposited on the surface, only 2D-extended islands were observed, each building-up of highly organized periodic protrusions, as shown in Figure 2.

In the case of PBA/Au(111) interface, repetitive unit consists of three pairs of disjoined protrusions (Figure 2a) rotated by 120°. In each domain, all repetitive units are rotated only clockwise or only anti-clockwise, leading to homochiral domains. The distance measured between the disjoined protrusions, is 0.8 ± 0.02 nm. For the CPBA molecules deposited on Au(111), a compact periodic network constituted by bright lines, which were separated by darker strips can be observed. The periodicity between the bright lines is 1.28 ± 0.02 nm. Each line is made up of paired bright protrusions separated by 0.8 ± 0.02 nm.

Based on our STM observations and measurements, we propose the models of PBA/Au(111) and CPAB/Au(111), respectively. Consistent with the features of PBA and CPBA molecules (Figure 1), each bright-paired protrusion is attributed to a single PBA or CPBA molecule respectively.

**Figure 3:**
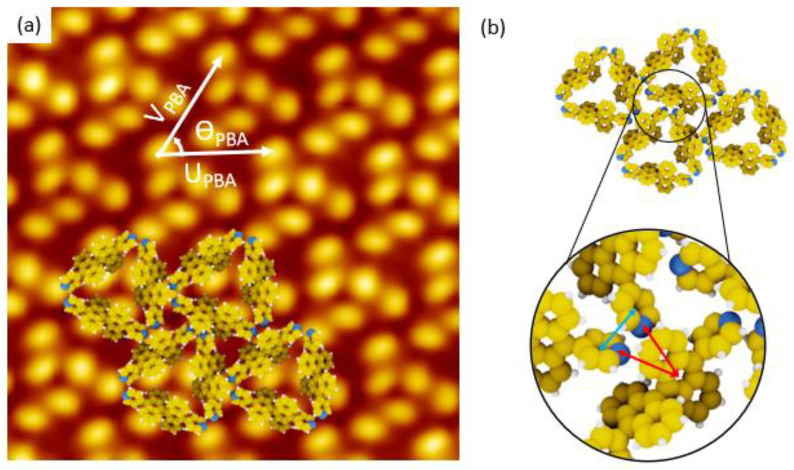
(**a**) STM image (Vs = −1.5 V, It = 10 pA, 8 nm × 8 nm) of PBA supramolecular network with corresponding superimposed model and unit cell vectors: U_PBA_ = 2.25 nm, V_PBA_ = 2.25 nm, ϴ_PBA_ = 60°. (**b**) Supramolecular model of PBA molecules on Au(111) surface with the inset of magnified image representing three adjacent PBA molecules, where two adjacent pyridyl rings are separated by a distance of 0.42 nm (blue arrow) and two nitrogen atoms point towards the centre of the anthracenyl ring of a third PBA molecule (highlighted by two red arrow, length: 0.41 nm).

In the case of PBA/Au(111) interface, the unit cell of the supramolecular network is constituted by three paired-bright protrusions included in a rhomb, as shown by the two vectors U_PBA_ and V_PBA_ (Figure 3a). The length of these two vectors are U_PBA_ = 2.25 ± 0.22 nm and V_PBA_ = 2.25 ± 0.22 nm. The unit cell covering 4.38 nm^2^ for three PBA molecules, the molecular density of the PBA/Au(111) network is 0.68 molecule per nm^2^. The supramolecular arrangement of PBA/Au(111) is obtained thanks to molecule-molecule interactions. The main molecule-molecule contribution is due to the interaction of nitrogen atoms of pyridyl groups pointing towards the centre of an anthracenyl ring of the adjacent PBA molecule (Figure 3b), corresponding to T-Shape π-π interaction [30].

**Figure 4:**
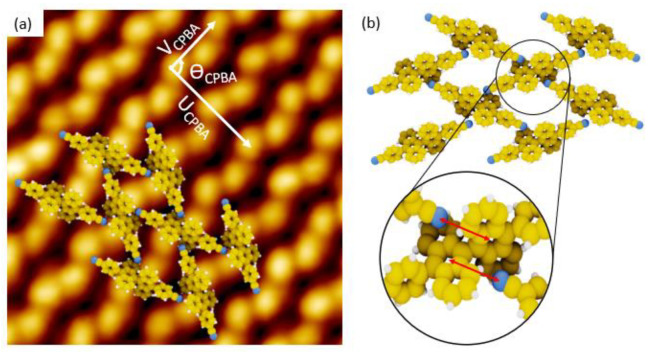
(**a**) STM image (Vs = 1.5 V, It = 10 pA, 8 nm × 8 nm) of CPBA supramolecular network with corresponded superimposed supramolecular model and unit cell vectors: U_CPBA_ = 2.58 nm, V_CPBA_ = 1.45 nm, ϴ_CPBA_ = 94°. (**b**) 3D model of CPBA/Au(111). Inset highlights the interaction between one nitrogen atom pointing out the center of anthracenyl ring of the adjacent molecule, which are separated by 0.45 nm (red arrow).

CPBA/Au(111) interface is described by an unit cell including two paired-bright protrusions. The unit cell is quite rectangular as shown by the vectors U_CPBA_ and V_CPBA_ (Figure 4a). The length of U_CPBA_ vector is 2.58 ± 0.26 nm while the length of V_CPBA_ vector is = 1.45 ± 0.15 nm. The U_CPBA_,V_CPBA_ angle is 94°. As the unit cell contains two PBA molecules for an area of 3.73 nm^2^, the molecular density of the CPBA/Au(111) network is 0.54 molecule per nm^2^, which is lower than those of PBA/Au(111) interface. This supramolecular network is supported by π-π interaction called T-shaped interaction [31] because the two nitrogen atoms of each CPBA molecule are pointed towards the centre of the anthracenyl ring of the adjacent CPBA molecule included in two surrounding lines of CPBA molecules (Figure 4b). In each unit cell, the two CPBA molecules are diastereo-isomer because of the relative orientation of their bianthryl core (Figure 4a).

### 3.2. Annealing of the Supramolecular Self-Assemblies on an Au(111) Surface

Then, we investigated the chemical transformation of the two supramolecular self-assemblies induced by thermal annealing. Hence, a set of experiments consisting of the subsequent annealing of each observed supramolecular network were conducted. From room temperature to 623 K, we did not observe any modification of the networks. However, at 623 K, a critical transformation was observed. Then, until 773 K, we did not observe other transformations but only noticeable desorption of formed nanostructures at 773 K. In addition, the duration of each step of annealing varied from 1 h to 4 h, but no effect of duration was observed.

**Figure 5:**
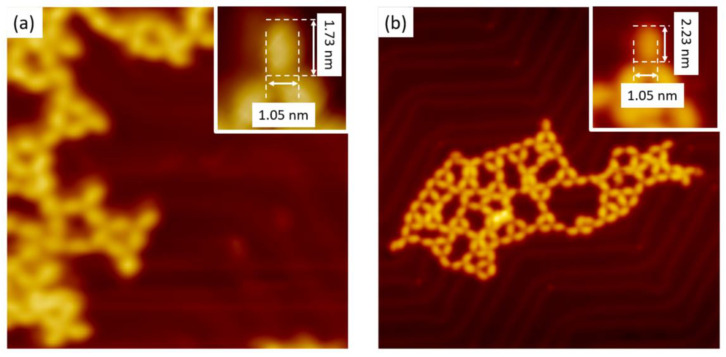
STM images of formed nanostructures observed on Au(111) surface after thermal annealing of supramolecular networks up to 673 K. (**a**) PBA (Vs = −1.7 V, It = 10 pA, 30 nm × 30 nm) inset 3 nm × 3 nm, (**b**) CPBA (Vs = −2.0 V, It = 7 pA, 60 nm × 60 nm, inset 3.5 nm × 3.5 nm).

The thermal annealing of the two periodic supramolecular networks described previously led to disordered annealing nanostructures (Figure 5). In addition, instead of bright-paired protrusions, nanostructures are constituted by cross-shaped bright protrusions. The length of these protrusions is 1.73 ± 0.05 nm and 2.23 ± 0.05 nm for PBA and CPBA respectively while their width is 1.05 ± 0.05 nm. The bright protrusions led to the formation of straight or reticulated nanolines including three- of four connecting nodes. The distance separating bright protrusions is longer in the case of CPBA than in the case of PBA (Figure 5).

## 4. Discussion

The on-surface behavior of bianthryl derivatives has been intensively investigated since it has been shown that most of this type of compound can be polymerized into graphene nanoribbons [7,11,28,32,33,34,35,36,37,38]. This polymerization occurs in two steps, the first being formation of a protopolymer and the second step of this process is based on surface-assisted intramolecular cyclodehydrogenation providing completely planar building blocks. This cyclodehydrogenation reaction occurs at around 550 K and 700 K on a Cu(111) and Au(111), respectively [33]. This difference of temperature outlines the role of the surface in this intramolecular cyclodehydrogenation. The driven force of cyclodehydrogenation is the formation of more conjugated compounds. One consequence of this increasing of conjugation is the flattening of involved molecules. However, on Cu(111), if the positions 10 and 10′ of the starting bianthryl are substituted by an aryl ring, the formation of protopolymers is not possible and only disordered polymeric structures were obtained due to the strong reactivity of the Cu(111) surface which lead to the formation of reactive radicals [18].

**Figure 6:**
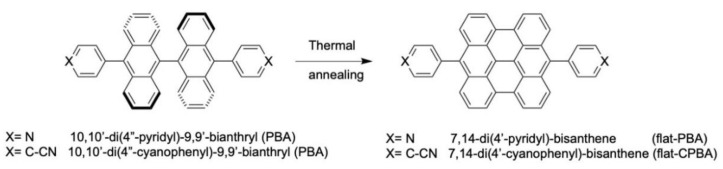
Thermal-induced cyclodehydrogenation of PBA and CPBA molecules. The starting PBA and CPBA molecules have a 3D cross-shaped core (i.e., bianthryl core), the corresponding bisanthene cores (Flat-PBA and flat-CPAB) are flattened.

On the basis of the features observed in STM images, we can attribute the bright protrusions (Figure 5) to the 7,14-(4′-pyridyl)-bisanthene (flat-PBA) and 7,14-(4′-cyanophenyl)-bisanthene (flat-CPBA) molecules obtained by surface-assisted cyclodehydrogenation of PBA and CPBA respectively (Figure 6).

As Au(111) surface is less reactive than Cu(111) surface and as the 10 and 10′-positions are substituted in PBA and CPBA molecules, no polymerisation occurs by thermal annealing [39]. Only intramolecular cyclodehydrogenations are possible, leading to flattened molecules, flat-PBA and flat-CPBA, respectively.

**Figure 7:**
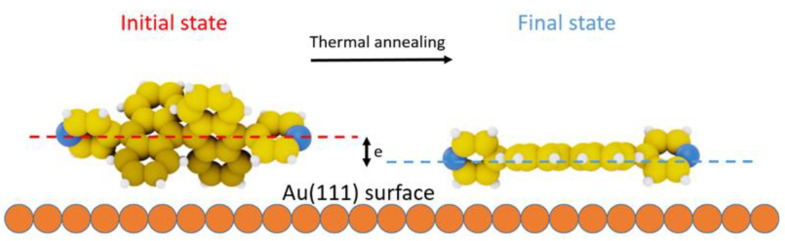
CPK model of PBA and flat-PBA highlighting the decreasing of molecule-surface distance by 0.24 nm due to the flattening of PBA induced by thermal annealing.

The average distance between Au(111) surface and flat-PBA or flat-CPBA molecules is shorter than those between surface and respectively, PBA and CPBA (Figure 7). Therefore, the interaction of nitrogen atoms of pyridyl or cyanophenyl moieties with the gold atoms of the surface is reinforced, that promotes the formation of coordination polymers [2,22,23,24,40,41,42].

The pyridyl or cyano moiety are ligand for several kind of metal atoms, due to the presence of the lone pair of electrons on the nitrogen atom. Due to the strength and the directionality of these metal-ligand bond, the corresponding coordination polymers exhibit a high degree of reticulation on metal substrates like on an Au(111) surface, even if it is still rare compared to other coinage surfaces [22,25,41,42]. These coordination properties support the observation of straight and reticulated nanolines in STM images.

**Figure 8:**
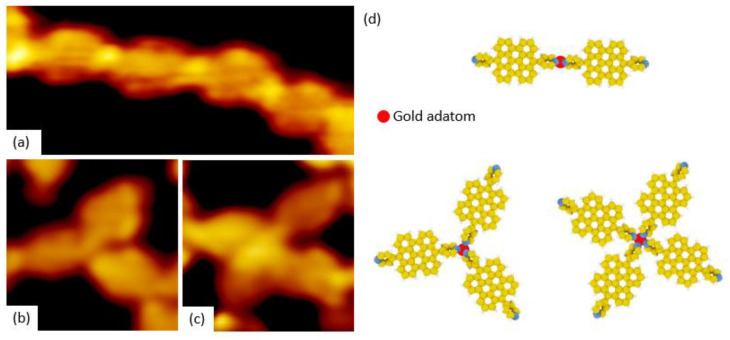
(**a**–**c**) STM images (Vs = −1.8 V, It = 10 pA) of PBA supramolecular nanostructures with three different configurations obtained after thermal annealing: (**a**) Chain-like nanostructures of protrusions (7 nm × 3 nm), (**b**) Y-shaped trimer nanostructures (4 nm × 4 nm) and (**c**) Cross-shaped of nanostructures (4 nm × 4 nm). (**d**) Molecular models of the three different type of coordination (red dot: Au ad-atom surrounded by organic moieties).

**Figure 9:**
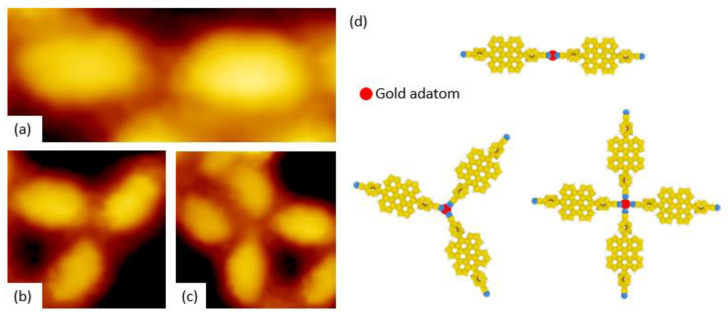
(**a**–**c**) STM images (Vs = −2.0 V, It = 7 pA) of three nanostructures obtained after thermal annealing of CPBA: (**a**) Rod-like protrusions (7 nm × 3 nm) (**b**) Y-shaped trimer of nanostructures (4 nm × 4 nm) and (**c**) Cross-shaped nanostructures (5 nm × 5 nm). (**d**) Molecular models of three different type of coordination (Red dot: Au ad-atom surrounded by organic moieties).

The straight lines observed in Figure 5 correspond to the coordination of two flat-PBA (Figure 8a,d) or flat-CPBA (Figure 9a,d) molecules surrounding a gold adatom, which is visible as bright protrusion for coordination polymers including flat-PBA. The three- and four reticulating nodes observed in STM images are attributed to three- or four flat-PBA (Figure 8b–d) or flat-CPBA molecules (Figure 9b–d) coordinating one gold adatom. Neither of these modes can form a long-range ordered lattice on Au(111) surface. The lack of long-range polymer can be explained by the softness of gold adatom compared to Co or Fe cations which lead to well-organized 1D/2D networks with hard nitrogen-based ligands [42].

## 5. Conclusions

We have demonstrated that the balance between molecule-molecule interaction and molecule-surface interaction can be strongly alter by using on-surface chemistry. We have shown that 2D-extended periodic supramolecular networks are converted into coordination polymers by a thermal annealing. By flattening molecule, thanks to intramolecular cyclodehydrogenations, pyridyl or cyanophenyl rings can be transformed from pi-pi stacking precursors to coordinating agents of gold adatoms. This strategy paves the way to new possibilities for multi-functional nanostructures by using on-surface assisted synthesis.
